# Geographic Distribution of Endemic Fungal Infections among Older Persons, United States

**DOI:** 10.3201/eid1802.111537

**Published:** 2012-02

**Authors:** Dirk Haselow, Mike Saccente, Keyur Vyas, Ryan Bariola, Haytham Safi, Robert Bradsher, Nate Smith, James Phillips

**Affiliations:** Arkansas Department of Health, Little Rock, Arkansas, USA (D. Haselow, H. Safi, N. Smith, J. Phillips);; University of Arkansas for Medical Sciences, Little Rock (D. Haselow, M. Saccente, K. Vyas, R. Bariola, R. Bradsher, N. Smith)

**Keywords:** endemic fungal infection, fungi, mycoses, coccidioidomycosis, histoplasmosis, blastomycosis, Arkansas

**To the Editor:** We read with interest the article by Baddley et al. (*1*) and appreciate their efforts to characterize incidence rates of mycoses. We agree that histoplasmosis, blastomycosis, and coccidioidomycosis are differential diagnoses for patients with consistent symptoms but who reside outside mycosis-endemic areas.

However, we believe that the methods of Baddley et al. probably do not determine the true incidence of these mycoses in sparsely populated states such as Arkansas. Their estimates contrast markedly with surveillance data from the Arkansas Department of Health (Table) and with our clinical experience as infectious disease physicians. We characterize Arkansas as a state in which histoplasmosis and blastomycosis incidence is high and coccidioidomycosis incidence is low; however, Baddley et al. indicate that in Arkansas, incidence of blastomycosis is relatively low and incidence of coccidioidomycosis is high.

**Table:** Reported cases of fungal diseases in Arkansas, by year*

Disease	No. cases/no. cases in persons >65 y of age											Incidence rate (95% CI)†	
	1999	2000	2001	2002	2003	2004	2005	2006	2007	2008		Overall	Persons >65 y
Blastomycosis, n = 166/43	20/5	15/3	20/9	15/3	19/6	17/4	16/6	13/3	15/1	16/3		4.3 (2.9–5.7)	1.1 (0–2.3)
Coccidioidomycosis, n = 3/0	2/0	0	0	0	0	1/0	0	0	0	0		0.08 (0–0.4)	0
Histoplasmosis, n = 372/65	15/3	13/3	23/6	22/2	16/4	42/9	51/9	66/4	78/13	46/12		9.6 (0–20)	1.7 (0–3.5)

*Data from Arkansas Department of Health. †No. cases/100,000 person-years, 1999–2008.

To investigate whether this finding might be associated with their small 5% sample of Medicare beneficiaries, we used data from the Arkansas census to determine that in 2008 the population of adults >65 years of age was ≈407,014, and during 1999–2008, there were ≈3,840,896 person-years for persons in this age group. A 5% sample would account for ≈192,045 person-years. Using their rate ranges (7.84–12.3 cases/100,000 person-years for histoplasmosis, 3.97–6.71 for coccidioidomycosis, and 0.39–0.86 for blastomycosis), we calculated the approximate numbers of cases in their sample: 15–23 histoplasmosis cases, 7–12 coccidioidomycosis cases, and only 1 blastomycosis case. Compared with rates from surveillance averaged over the 10 years, the midpoints of the Baddley et al. estimates are ≈6-fold higher for histoplasmosis, ≈60-fold higher for coccidioidomycosis, and ≈0.4-fold lower for blastomycosis. Only their estimate for blastomycosis incidence falls within the 10-year 95% CIs from surveillance data. We believe that the small cell sizes require that the rate estimates of Baddley et al. be interpreted with care, especially with respect to less populous states.
