# Supplementary material for: Geographic Distribution of Endemic Fungal Infections among Older Persons, United States
Source: Emerg Infect Dis. 2012 Feb;18(2):360–1. doi: 10.3201/eid1802.111537 (PMC3311154; doi:10.3201/eid1802.111537)
Supplement: Related texts

# Reply: Geographic Distribution of Endemic Fungal Infections among Older Persons, United States

**DOI:** 10.3201/eid1802.111617

**Authors:** John W. Baddley, Fenglong Xie, Jeffrey R. Curtis

**Affiliations:** University of Alabama at Birmingham, Birmingham, Alabama, USA

**In Response:** We thank Haselow et al. (*1*) for their careful review of our article (*2*). They raise the relevant concern about potential instability of incidence rates from our data because of small cell sizes. We agree that use of administrative data has major limitations. As such, our intent was not to compare infection incidences of individual states; but rather, our intent was to focus on geographic distribution of endemic mycoses and whether infections occurred in non–mycosis-endemic areas.

Specifically, for blastomycosis, our study showed incidence in Arkansas to be 0.8 (0.12–5.8) cases per 100,000 person-years, comparable to the rate provided by Haselow et al. of 1.1 case per 100,000 person-years (*1*). For coccidioidomycosis, our study found the rate to be much higher than that calculated from the Arkansas surveillance data. Potential reasons for this discrepancy might be lack of case capture with surveillance data, because mandatory reporting for coccidioidomycosis is not required in Arkansas, or misclassification of incident cases in the administrative data. Finally, for histoplasmosis, the incidence rate calculated from administrative data was much higher than that reported by Haselow et al. By using administrative data, we identified a large number (15) of cases and doubt that rate instability is present. We agree that surveillance that uses administrative data has inherent limitations, which require that care be taken when interpreting epidemiologic measures, especially when sample sizes are small.

## References

[1] Haselow D, Saccente M, Vyas K, Bariola R, Safi H, Bradsher R, Geographic distribution of endemic fungal infections among older persons, United States [letter]. Emerg Infect Dis. 2012;18:361 .10.3201/eid1802.111537PMC331115422305518

[2] Baddley JW, Winthrop KL, Patkar NM, Delzell E, Beukelman T, Xie F, Geographic distribution of endemic fungal infections among older persons, United States. Emerg Infect Dis. 2011;17:1664–9. 10.3201/eid1709.10198721888792PMC3322071
